# Vapor growth of V-doped MoS_2_ monolayers with enhanced B-exciton emission and broad spectral response

**DOI:** 10.1007/s12200-023-00097-w

**Published:** 2023-12-07

**Authors:** Biyuan Zheng, Xingxia Sun, Weihao Zheng, Chenguang Zhu, Chao Ma, Anlian Pan, Dong Li, Shengman Li

**Affiliations:** 1 Key Laboratory for Micro-Nano Physics and Technology of Hunan Province, State Key Laboratory of Chemo/Biosensing and Chemometrics, Hunan Institute of Optoelectronic Integration, College of Materials Science and Engineering, Hunan University, Changsha, 410082 China; 2 College of Advanced Interdisciplinary Studies and Hunan Provincial Key Laboratory of Novel Nano Optoelectronic Information Materials and Devices, National University of Defense Technology, Changsha, 410073 China; 3 Wuhan National Laboratory for Optoelectronics (WNLO), Huazhong University of Science and Technology (HUST), Wuhan, 430074 China

**Keywords:** Atomic substitution, V-doped MoS_2_, Distinct B-exciton, Broad spectral response

## Abstract

Dynamically engineering the optical and electrical properties in two-dimensional (2D) materials is of great significance for designing the related functions and applications. The introduction of foreign-atoms has previously been proven to be a feasible way to tune the band structure and related properties of 3D materials; however, this approach still remains to be explored in 2D materials. Here, we systematically demonstrate the growth of vanadium-doped molybdenum disulfide (V-doped MoS_2_) monolayers via an alkali metal-assisted chemical vapor deposition method. Scanning transmission electron microscopy demonstrated that V atoms substituted the Mo atoms and became uniformly distributed in the MoS_2_ monolayers. This was also confirmed by Raman and X-ray photoelectron spectroscopy. Power-dependent photoluminescence spectra clearly revealed the enhanced B-exciton emission characteristics in the V-doped MoS_2_ monolayers (with low doping concentration). Most importantly, through temperature-dependent study, we observed efficient valley scattering of the B-exciton, greatly enhancing its emission intensity. Carrier transport experiments indicated that typical p-type conduction gradually arisen and was enhanced with increasing V composition in the V-doped MoS_2_, where a clear n-type behavior transited first to ambipolar and then to lightly p-type charge carrier transport. In addition, visible to infrared wide-band photodetectors based on V-doped MoS_2_ monolayers (with low doping concentration) were demonstrated. The V-doped MoS_2_ monolayers with distinct B-exciton emission, enhanced p-type conduction and broad spectral response can provide new platforms for probing new physics and offer novel materials for optoelectronic applications.

**Graphical abstract:**

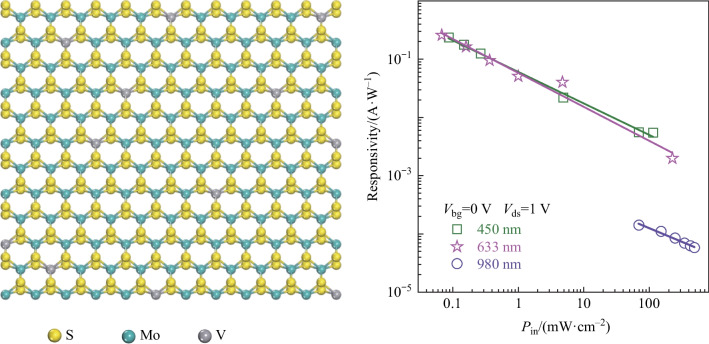

**Supplementary Information:**

The online version contains supplementary material available at 10.1007/s12200-023-00097-w.

## Introduction

Two-dimensional (2D) semiconductors have attracted attention due to their future application in field effect transistors [1–6], photodetectors [7–10], laser [11, 12], photoelectrochemical [13], and other devices [14, 15]. Molybdenum disulfide (MoS_2_), as a typical 2D semiconductor, has direct band gap (~ 1.8 eV) [16–18] and high mobility [1, 5], and shows great potential in optoelectronic devices [1–3, 5, 8, 14]. Due to the broken inversion symmetry, inducing the spin–orbit coupling, there is a large splitting of ~ 160 meV over the valence band in monolayer MoS_2_, giving rise to A- and B-excitons [16, 18–20]. Generally, A-exciton behavior dominates the emission of the MoS_2_, while observing B-exciton behavior is difficult due to the forbiddance of its transition [17, 21]. Meanwhile, achieving p-type conductivity in chemical vapor deposition (CVD) grown MoS_2_ monolayer is challenging because of its insurmountable sulfur vacancy defect [22, 23]. Furthermore, due to the large band gap, photodetectors based on MoS_2_ monolayers show a limited detection range in visible light [8]. Consequently, effectively manipulating the photoelectric properties of MoS_2_ and further enriching its properties are important for both fundamental research and applications.

Foreign substitutional doping is efficacious, stable, and exhibits long-range ordering, making it a preferred method for altering the electronic band structure, conduction type, and carrier concentration of pristine materials. Previous reports suggested that vanadium (element V) is an attractive dopant for room-temperature ferromagnetism and has found widespread use in many magneto-optic devices [24–27]. The introduction of V element in MoS_2_ offers an effective means to modulate both the optical and the electrical properties of the samples, potentially leading to new applications and functionalities. Very recently, CVD growth of V-doped MoS_2_ for use in synaptic transistors and quasi-continuously tunable carrier polarity transistors have been reported [28–30]. However, to the best of our knowledge, the A- and B-excitons properties and the photoelectric applications of V-doped MoS_2_ have not been systematically studied.

In this work, V-doped MoS_2_ monolayers were grown using an alkali metal-assisted CVD approach. The achievement of V doping in high-quality crystalline monolayers were confirmed by scanning transmission electron microscopy (STEM), X-ray photoelectron spectroscopy (XPS) and Raman spectra. The emergence of p-type conduction in the achieved samples further demonstrated the successful introduction of V atoms into the crystal structure. What’s even more interesting is, the distinct B-exciton emission, enhanced p-type conduction and broad spectral response observed in the V-doped MoS_2_. Our results could offer a novel approach to modulating exciton properties in 2D semiconductors and potentially trigger numerous applications in spintronics.

## Experimental

### Materials synthesis

A one-zone furnace was used for growth of V-doped MoS_2_ monolayers. Firstly, one quartz boat (boat 1), loaded with sublimate sulfur powder (S), was placed at the upstream, while another (boat 2), containing a mixture of molybdenum trioxide (MoO_3_, Alfa Aesar, 99%), vanadium pentoxide (V_2_O_5_, Alfa Aesar, 99%), and potassium iodide (KI, Alfa Aesar, 99%), was positioned at the center of a quartz tube (25 cm in diameter and 100 cm long). A long piece of SiO_2_/Si (270 nm SiO_2_, 1 cm $\times$ 3 cm) substrate was placed above the quartz boat 2. The quartz tube containing the reaction sources and the deposited substrate was placed in the furnace, and the quartz boat 2 was ensured to be located in the center of the furnace. High purity Ar was introduced from the upstream as carrier gas and discharged from the downstream of the quartz tube. Prior to heating, high-rate Ar gas flow (1000 SCCM) was used to remove any active gases. The furnace was then heated to 750–800 ℃ in 30 min and held at that temperature for 5−10 min to grow the V-doped MoS_2_ monolayers. Throughout the growth process, the Ar gas flow was maintained at 50 SCCM. Subsequent to growth, the furnace was then allowed to cool naturally to room temperature.

### Characterizations

*Raman and photoluminescence* (PL)*.* The Raman/PL was performed using a μ-PL system (WITec, alpha-300) equipped with a 532 nm argon ion laser.

*Atomic force microscope* (AFM). The AFM observations were carried out using a Bruker Dimension ICON instrument.

*Scanning transmission electron microscopy*. The STEM observations were acquired using a JEOL ARM200F microscope.

### Device fabrication and measurements

Source-drain electrodes of the back-gated V-doped MoS_2_ devices were fabricated using a transfer method. Au with a thickness of 50 nm was prepared on a bare SiO_2_/Si substrate using photolithography and thermal evaporation deposition methods and subsequently transferred onto the top of the V-doped MoS_2_ monolayers as source/drain. The optoelectrical properties of the V-doped MoS_2_ devices were measured using Lake Shore Probe Station and Agilent B1500A semiconductor analyzer.

## Results and discussion

**Fig. 1:**
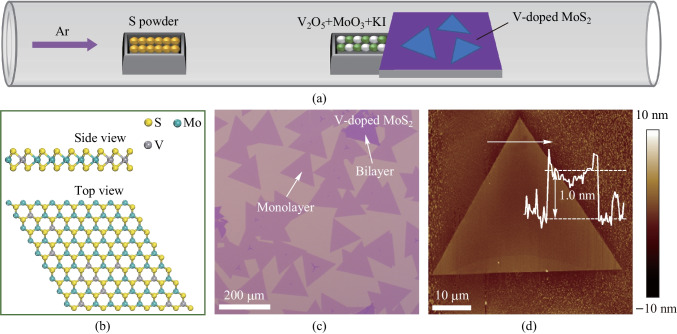
Schematic of vapor growth and AFM characterization of V-doped MoS_2_ monolayers. **a** Schematic of the synthesis process of V-doped MoS_2_ monolayers. **b** Side- and top-views of the crystal structure of V-doped MoS_2_ monolayer. The yellow, blue and gray spheroids represent S, Mo and V atoms, respectively. **c** Optical image of the V-doped MoS_2_ monolayers. **d** AFM image of the V-doped MoS_2_ monolayer. Inset: height profile of the V-doped MoS_2_ measured along white arrow in (**d**), indicating that the thickness of detected sample is about 1.0 nm

V-doped MoS_2_ monolayers were synthesized using an alkali metal-assisted CVD method as depicted in Fig. 1a. As is the case in previous reports, the chemical reaction between MoO_3_, V_2_O_5_ and the KI results in formation of volatile oxyhalide species [31, 32]. In brief, solid-phase MoO_3_ reacts with solid-phase KI, producing the gaseous-species MoO_2_I_2_. Similarly, the solid-phases V_2_O_5_ and KI react with each other to form gaseous-phase VOI_3_. The gas–gas phase reactions among MoO_2_I_2_, VOI_3_ and S could occur easily and efficiently. The possible reaction routes in the system are listed as follows:

1 $${2\mathrm{MoO}}_{3}\left(\mathrm{s}\right) + 2\mathrm{KI}\left(\mathrm{s}\right) \to {\mathrm{K}}_{2}{\mathrm{MoO}}_{4}\left(\mathrm{s}\right) +\mathrm{ Mo}{\mathrm{O}}_{2}{\mathrm{I}}_{2}\left(\mathrm{g}\right)$$

2 $${{2\mathrm{V}}_{2}\mathrm{O}}_{5}\left(\mathrm{s}\right) + 3\mathrm{KI}\left(\mathrm{s}\right) \to 3{\mathrm{KVO}}_{3}\left(\mathrm{s}\right) +\mathrm{ VO}{\mathrm{I}}_{3}\left(\mathrm{g}\right)$$

3 $$2\left(1-x\right){\mathrm{MoO}}_{2}{\mathrm{I}}_{2}\left(\mathrm{g}\right) + 2x{\mathrm{VOI}}_{3}\left(\mathrm{g}\right) + 3\mathrm{S}\left(\mathrm{g}\right) \to {2\mathrm{V}}_{x}{\mathrm{Mo}}_{1-x}{\mathrm{S}}_{2}\left(\mathrm{S}\right) + \left(2-x\right){\mathrm{SO}}_{2}\left(\mathrm{g}\right) + {\left(2+x\right)\mathrm{I}}_{2}\left(\mathrm{g}\right)$$

Figure 1b displays the side- and top-views of the atomic structure of the V-doped MoS_2_ monolayer, wherein the yellow, blue and gray spheroids represent the S, Mo and V atoms, respectively. V atoms have substituted and occupied the sites of Mo atoms. Figure 1c shows the optical image of the V-doped MoS_2_ monolayers, indicating the uniform distribution on a large scale (> 100 μm). Additionally, though in a small proportion, V-doped MoS_2_ bilayers with deeper contrast than monolayers were observed and are marked in Fig. 1c. AFM characterization was used to confirm the thickness of the V-doped MoS_2_ nanosheet (Fig. 1d), which was measured to be about 1.0 nm (inset in Fig. 1d), indicating a single layer nature.

**Fig. 2:**
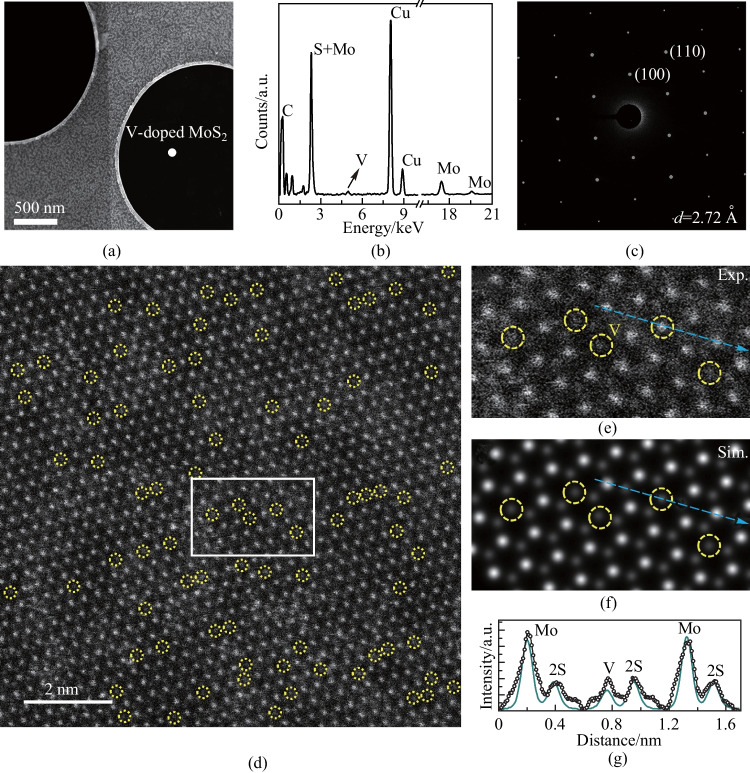
Structural and chemical composition of V-doped MoS_2_ monolayer. **a** Low-magnification ADF STEM image of the V-doped MoS_2_ monolayer. **b** TEM–EDX profile collected from white dot in (**a**), shows the V mole fraction (*x*) is 0.11. **c** SAED pattern of the V-doped MoS_2_ monolayer along the zone axis of [0001]. **d** HRSTEM image of the V-doped MoS_2_ monolayer, the V atoms are highlighted by yellow dotted circles. **e**, **f** Enlarged, **e** experimental, and **f** simulation HRSTEM images of the yellow rectangle region in (**d**). **g** Electron intensity profile along the blue arrows in (**e**) and (**f**)

The atomic structure and chemical composition of the V-doped MoS_2_ monolayers were investigated by STEM, energy dispersive X-ray spectroscopy (EDS), high-resolution STEM (HRSTEM) and selected-area electron diffraction (SAED). Figure 2a displays a typical low-magnification annular dark-field (ADF) STEM image of a single-domain V-doped MoS_2_ sample transferred onto a Cu grid. In Fig. 2b, the EDS spectrum obtained from the white dot in Fig. 2a revealed elemental peaks corresponding to S, Mo, and V, confirming the composition of the doped nanosheets. Thus, we could deduce the V mole fraction of the V-doped MoS_2_ sample, indicating a composition of V_0.11_Mo_0.89_S_2_. Figure S1 shows the EDS spectra taken from more samples, these samples were used for Raman, PL and electrical characterizations.

Figure 2c presents the SAED pattern of the sample, displaying a single set of diffraction spots with sixfold symmetry, confirming the single-crystal nature of V-doped MoS_2_. The lattice spacing of the V-doped MoS_2_ was obtained using the diffraction peaks yields (100) and was measured to be 0.272 nm, which is similar with that of MoS_2_ [5]. HRSTEM was employed to characterize the atomic structure of the achieved V-doped MoS_2_ (Fig. 2d), in which the different atoms could be identified using Z-contrast (Z^2^_Mo_ = 1764, Z^2^_V_ = 529 and Z^2^_S_ + Z^2^_S_ = 512). Figure 2e and f provide enlarged experimental and simulation HRSTEM images, in which V atoms are marked with yellow dotted circles. As shown in Fig. 2g, the ADF intensity profile acquired along the blue arrow in Fig. 2e and f also confirms similar results.

**Fig. 3:**
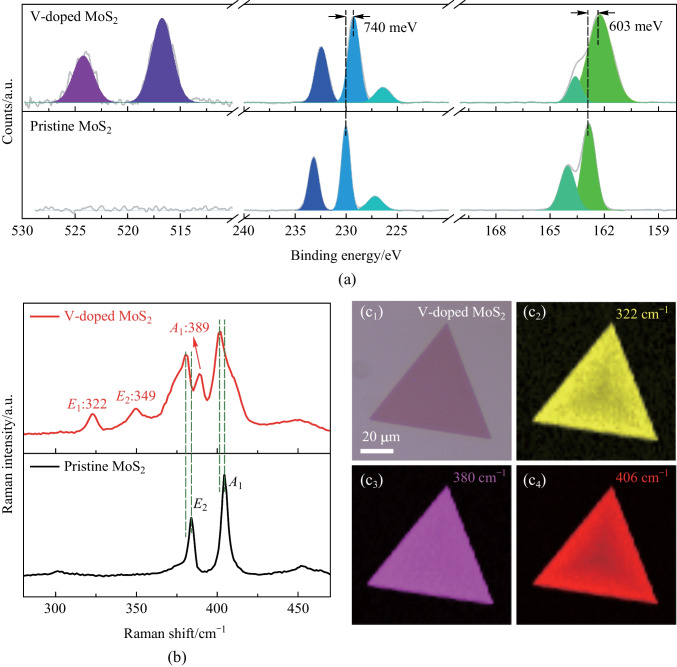
XPS and Raman characterizations of V-doped MoS_2_ monolayers. **a** XPS spectra of the V-doped MoS_2_ (upper half) and pristine MoS_2_ (lower half) monolayers, respectively. **b** Raman spectra of the V-doped MoS_2_ (upper half) and pristine MoS_2_ (lower half) monolayers, respectively. **c**_**1**_ Optical and **c**_**2**_−**c**_**4**_ Raman intensity mapping images of the selected V-doped MoS_2_ monolayer

XPS was adopted to characterize the chemical states of elements in another V-doped MoS_2_ sample. From the XPS survey spectra as shown in Fig. S2, we determined that the V mole fraction (*x*) of the tested samples was 9%, indicating a composition of V_0.09_Mo_0.91_S_2_. Figure 3a shows the element binding energy comparison between V-doped MoS_2_ (upper half) and pristine MoS_2_ (lower half). For MoS_2_, five peaks were observed at 233.2, 230.0, 227.2, 164.0 and 162.9 eV. The first two peaks are attributed to the Mo element (Mo^4+^ 3d_3/2_ and Mo^4+^ 3d_5/2_), while the remaining peaks belong to S element (S^2−^ 2s, S^2−^ 2p_3/2_ and S^2−^ 2p_3/2_) [33]. In the case of V-doped MoS_2_, a slight down-shift of the above peaks was observed. Such down-shift indicated that the Fermi level of the achieved V-doped MoS_2_ was closer to the valence band compared with that of the pristine MoS_2_ monolayer, which demonstrated an increase in p-type doping concentration [34, 35]. Moreover, two additional peaks located at 516.7 and 524.3 eV were observed, which was attributed to the presence of V atoms. These two peaks were indexed as V^4+^ 2p_3/2_ (516.7 eV) and V^4+^ 2p_1/2_  (524.3 eV) [36].

Raman spectra were used to investigate the vibration modes of the V-doped MoS_2_ monolayers (V_0.11_Mo_0.89_S_2_), while pristine MoS_2_ was chosen as a representative sample. Pristine MoS_2_ exhibited two Raman peaks centered at 384 and 404 cm^−1^, corresponding to the *E*_2g(S-Mo)_ mode and the *A*_1g(S-Mo)_ mode, respectively (bottom panel in Fig. 3b). However, in the Raman spectrum collected from the V-doped MoS_2_ monolayer (top panel in Fig. 3b), four additional peaks located at 323, 349, 389 and 406 cm^−1^ were observed, indicating the introduction of V atoms [37, 38]. It is worth noting that, compared with pristine MoS_2_, the *E*_2g(S-Mo)_ and *A*_1g(S-Mo)_ peaks taken from V-doped sample exhibited a slight down-shift of 2.82 and 2.96 cm^−1^, respectively, further implying hole doping induced by V atoms [34]. Raman mapping images of the V-doped MoS_2_ monolayer (as shown in Fig. 3c) demonstrated the uniform chemical composition of the tested sample.

**Fig. 4:**
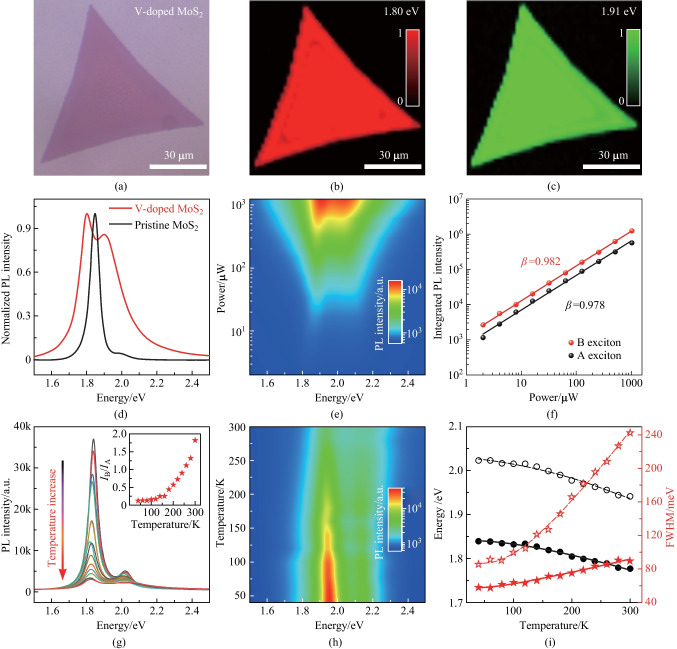
Optical characterization of V-doped MoS_2_ monolayers. **a** Optical image and **b**, **c** PL intensity mapping images of the V-doped MoS_2_ monolayer at **b** 1.80 eV and **c** 1.91 eV, respectively. **d** Normalized PL spectra of the pristine MoS_2_ (black line) and V-doped MoS_2_ (red line) monolayers. **e** 2D pseudocolor plot of the emission spectra under illumination by the laser excitation power densities from 2 to 1240 μW. **f** Integrated PL intensities of the A- and B-excitons. **g** PL spectra from the V-doped MoS_2_ monolayer from 40 to 300 K. Insets: presents the emission intensities ratio ${\eta }_{\mathrm{P}}$ between the A- and B-excitons at different temperatures. **h** 2D pseudocolor plots of temperature-dependent PL spectra of the V-doped MoS_2_ monolayer. **i** Corresponding optical band gap and PL FWHM as a function of temperature together with their fits

The optical properties of the V-doped MoS_2_ monolayers were investigated with steady-state PL spectroscopy. Figure 4a shows the optical image of a selected V-doped MoS_2_ monolayer with low doping concentration (V_0.05_Mo_0.95_S_2_). The typical PL spectrum collected from pristine MoS_2_ (depicted by a black line in Fig. 4d) exhibited two peaks centered at 1.85 and 2.00 eV, corresponding to A- and B-excitons, respectively. Clearly, the A-exciton emission dominated the whole emission behavior. The ratio of the emission integrated intensities between the B- and A-excitons was calculated as ${\eta }_{\mathrm{P}}={I}_{\mathrm{B}}/{I}_{\mathrm{A}}=0.083$, where *I*_A_ and *I*_B_ are the integrated intensities of A- and B-excitons, respectively. In contrast, the spectrum of the V-doped MoS_2_ (illustrated by a red line in Fig. 4d) showed distinct B-exciton emission that did not occur in the case of pristine MoS_2_. The ${\eta }_{\mathrm{P}}$ of the V-doped MoS_2_ was calculated to be 3.13, which was 38 times higher than that occurring in the case of pristine MoS_2_ monolayer. In addition, noticeable red-shifts of both the emission peaks of A- (~ 46 meV) and B-excitons (~ 99 meV) were observed in the V-doped MoS_2_ monolayer, primarily resulting from doping-induced bandgap narrowing [39]. The split of A- and B-exciton bands (${\Delta }_{\mathrm{BA}}={E}_{\mathrm{B}}-{E}_{\mathrm{A}}$) was determined to be ~ 110 meV for V-doped MoS_2_ monolayer, smaller than that of pristine MoS_2_ monolayer of ~ 150 meV, indicating that the doped V atoms effectively reduced the spin–orbit splitting between A- and B-excitons behavior in MoS_2_ monolayer (Fig. S3). As shown in Fig. S4, for the V_0.02_Mo_0.98_S_2_ and V_0.05_Mo_0.95_S_2_ monolayers (with low doping concentration), the ${\eta }_{\mathrm{P}}$ increased with the increase of V composition. But the sample with high doping concentration (V_0.11_Mo_0.89_S_2_) did not have PL emission. Such PL quenching is contributed to the localized exciton trapped states caused by doped V atoms (Fig. S3) [39].

Figure 4b and c show the energy-selected PL intensity mapping images of the V-doped MoS_2_ at 1.80 and 1.91 eV, respectively. The slightly non-uniform PL mapping is mainly caused by strain effects [40]. To further explore the spectral features, we studied the excitation power dependence of A- and B-excitonic transitions. Figure 4e presents the 2D pseudocolor plots of PL spectra under illumination by different laser excitation power densities. The log–log plots of integrated PL intensities of the A- (black dots in Fig. 4f) and B-excitons (red dots in Fig. 4f), as functions of excitation power, are presented in Fig. 4f. The relationship between integrated PL intensity *I* and excitation power *P* can be written as: *I* = *P*^*β*^, where *β* ≈ 1 implies an exciton-like transition [41]. Temperature-dependent experiments were conducted to explore the distinct B-exciton emission. Figure 4g exhibits the PL spectra measured in the V-doped MoS_2_ monolayer at different temperatures, while the corresponding 2D pseudocolor results are shown in Fig. 4h. The inset in Fig. 4g presents the integrated intensities ratio (${\eta }_{\mathrm{P}}$) of A- and B-excitons at different temperatures. As can be seen, ${\eta }_{\mathrm{P}}$ increased gradually with rising temperature, indicating the activation of B-exciton emission was at high temperature. This can be attributed to the enhancement of efficient valley scattering with the assistance of phonons (Fig. S3) [42]. On the contrary, the abnormal behavior here was attributed to the efficient valley scattering induced by the V atoms doping, which should be largely suppressed at low temperature, subsequently quenching the B-exciton emission.

In addition, we also studied the exciton-phonon interactions of the V-doped MoS_2_ monolayer. As can be seen, the two emission peaks broadened and shifted towards lower energy with increasing the temperature (Fig. 4i). Such variation can be well interpreted in terms of by Varshni’s semi-empirical formula:

4 $${E}_{\mathrm{g}}\left(T\right) ={ E}_{\mathrm{g}}\left(0\right) - \frac{\alpha {T}^{2}}{T + \beta },$$

where *α* and *β* are the characteristics of the given material. We found that the energies shift of A- and B-excitons of the V-doped MoS_2_ matched well Eq. (4), and the results are shown in Table 1. Such observed unequal shifts of A- and B-excitons were quite similar to those of other TMDCs [42].

**Table 1:** Fitting results of PL energies shift at different temperatures

Exciton	*E*_g_(0)/eV	*α*/(eV ·K^−1^)	*β*/K
A-exciton	1.843 ± 0.002	(6.258 ± 2.595) × 10^−4^	514.83 ± 341.89
B-exciton	2.027 ± 0.003	(8.815 ± 4.420) × 10^−4^	566.25 ± 438.83

Full width variation at half-maximum (FWHM) of these spectra is shown in Fig. 4i, which can be described by Bose−Einstein type expression:

5 $$\Gamma \left(T\right) = {\Gamma }_{0} + \frac{{\Gamma }_{\mathrm{LO}}}{{\mathrm{e}}^{{\omega }_{\mathrm{LO}}/(kT)} - 1},$$

where Γ_0_ represents the inhomogeneous broadening term and the line width at 0 K, Γ_LO_ represents the exciton-longitudinal optical (LO) phonon coupling, and the *ω*_LO_ is the dominant phonon or an average phonon energy. The values of the parameters are summarized in Table 2. The temperature-dependent shifts of the energies and FWHM of the excitonic transitions are mainly due to the lattice constant variations and interactions with relevant acoustic and optical phonons [43].

**Table 2:** Fitting results of PL FWHM at different temperatures

Exciton	Γ_0_/meV	Γ_LO_/meV	*ω*_LO_/eV
A-exciton	57.523 ± 1.216	37.036 ± 11.762	25.616 ± 6.044
B-exciton	86.375 ± 2.108	313.684 ± 39.271	38.074 ± 2.838

**Fig. 5:**
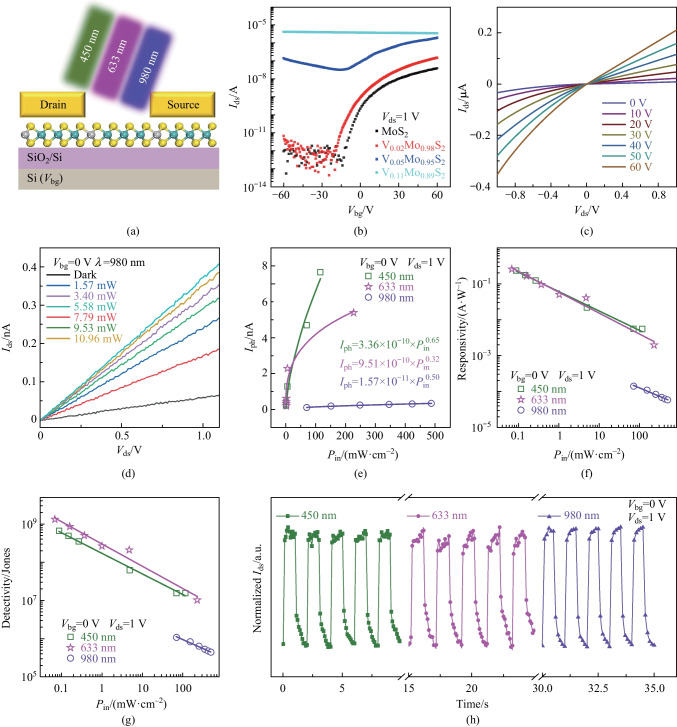
Electrical transport and photoelectric properties of V-doped MoS_2_ monolayers. **a** Device schematic diagram of V-doped MoS_2_. **b** Transfer characteristics of the MoS_2_, V_0.02_Mo_0.98_S_2_, V_0.05_Mo_0.95_S_2_ and V_0.11_Mo_0.89_S_2_ monolayers, showing the transfer behavior from n- to ambipolar to p-type, respectively. **c** Output characteristics of the V_0.02_Mo_0.98_S_2_ transistor. **d** *I*_ds_ − *V*_ds_ curves of the V-doped MoS_2_ device measured in dark and under illumination by 980 nm laser with different incident power densities. **e** Photocurrent, **f** responsivity and **g** detectivity as a function of illumination power density under illumination by 450, 633 and 980 nm lasers, respectively. **h** Time-resolved photoresponse of the V-doped MoS_2_ device under illumination by 450, 633 and 980 nm lasers

To explore the electronic properties and applications of our achieved V-doped MoS_2_ monolayers, we fabricated back-gated field-effect transistors. Figure 5a provides the schematic diagram of the device. Figure 5b shows the transport properties of the samples with different V composition (MoS_2_, V_0.02_Mo_0.98_S_2_, V_0.05_Mo_0.95_S_2_ and V_0.11_Mo_0.89_S_2_). It is evident that both the MoS_2_ and V_0.02_Mo_0.98_S_2_ exhibited typical n-type unipolar transfer behavior. With the introduction of V atoms, transitions from n-type first to ambipolar and then to p-type conduction occurred, revealing that the V atom doped in MoS_2_ monolayer could effectively implement p-type doping. Ambipolar conduction behavior was observed in V_0.05_Mo_0.95_S_2_ based device, while p-type unipolar conduction behavior was obtained with further increases in the V doping concentration (V_0.11_Mo_0.89_S_2_), which was consistent with the Raman and XPS results. The above results demonstrated that with the increase of V component, the V-doped MoS_2_ exhibited a transition from non-degenerate (MoS_2_ and low doping concentration V-doped MoS_2_) to degenerate (high doping concentration V-doped MoS_2_) semiconductor.

The photoelectric behavior of the V-doped MoS_2_ was then examined and V_0.02_Mo_0.98_S_2_ monolayer based device was selected. Figure 5c shows the output characteristic curves, exhibiting liner results. Figure S5 displays the photoconductive properties of the V-doped MoS_2_ monolayer device under illumination by 450 and 633 nm lasers with different power densities. It is clear that, the channel current increased gradually with increases in the power densities (*P*_in_), which demonstrated that the V-doped MoS_2_ monolayer has good visible light response. It is worth noting that our V-doped MoS_2_ monolayer device exhibited significant light response under illumination by near infrared light at a wavelength of 980 nm; the *I*_ds_–*V*_ds_ curves are shown in Fig. 5d. The obtained extracted photocurrents (${I}_{\mathrm{ph}}={I}_{\mathrm{light}}-{I}_{\mathrm{dark}}$) under illumination by different laser power are shown in Fig. 5e, fitted with an equation of ${I}_{\mathrm{ph}}=a{P}^{\alpha }$. The fitted parameter of $a$ were 3.36 × 10^–10^, 9.51 × 10^−10^ and 1.57 × 10^−11^; and *α* were 0.65, 0.32 and 0.50 under illumination by 450, 633 and 980 nm lasers, respectively. The responsivity (*R*) was calculated by the equation of $R={I}_{\mathrm{ph}}/(P\times A)$, where ${I}_{\mathrm{ph}}$ is the photocurrent, *P* is the incident light power density, and *A* is the effective area of the device channel (*A* = 1207.44 μm). As shown in Fig. 5f, the responsivity of the device based on V-doped MoS_2_ monolayer reached up to 0.23, 0.25 and 1.41 × 10^−4^ A/W under illumination by 450, 633 and 980 nm lasers, respectively. Detectivity (D*) is used to characterize the sensitivity of a photodetector, which can be estimated by ${D}^{*}=R{A}^{1/2}/{(2\mathrm{e}\times {I}_{\mathrm{dark}})}^{1/2}$. The maximum estimated D* values were 6.59 × 10^8^, 1.31 × 10^9^ and 1.07 × 10^6^ Jones under illumination by 450, 633 and 980 nm lasers, respectively (Fig. 5g). The summarized result of the responsivity and detectivity is shown in Table 3. The performance comparison between the photodetectors based on V-doped MoS_2_ monolayer and other reported photodetectors is shown in Table S1, which demonstrates that our photodetectors have moderate performance [44–52]. Figure 5h shows the switching behavior of the photodetector measured under light irradiation with visible light 450 (green line), 633 (red line) and near-infrared light 980 nm (violet line) lasers, indicating that the device could be switched effectively between high resistance and low resistance states. As shown in Fig. S3, in V-doped MoS_2_ monolayers, an accept band lever caused by V doping between the conduction band and valence band can be introduced, which can facilitate the absorption of short-wavelength light, enabling near-infrared light detection. The above results indicate that the V-doped MoS_2_ device exhibits significant broad spectral response.

**Table 3:** Performances of the V-doped MoS_2_ photodetectors under different laser illumination

Laser wavelength/nm	Responsivity at 1 V/(A⋅W^−1^)	Detectivity/Jones
450	0.23	6.59 × 10^8^
633	0.25	1.31 × 10^9^
980	1.41 × 10^−4^	1.07 × 10^6^

## Conclusion

In summary, a series of substitutional V-doped MoS_2_ monolayers were synthesized using an alkali metal-assisted CVD growth method. The samples were systematically characterized by XPS, Raman, STEM and electrical transport characterizations, confirming that V atoms were uniformly doped into the MoS_2_ samples. Interestingly, enhanced B-exciton emission was observed in the doped samples and the emission behaviors were systematically studied using steady temperature-dependent PL experiments. Electrical transport measurements indicated that enhanced p-type conduction occurred in the achieved V-doped MoS_2_ monolayers. Moreover, the photodetector based on V-doped MoS_2_ monolayer showed broad spectral response from visible to near-infrared light. The synthesized V-doped MoS_2_ nanosheets can provide new material platform for spintronics related fundamental research and device applications.

### Supplementary Information

Below is the link to the electronic supplementary material.

Supplementary file1 (PDF 587 KB)

## Data Availability

The data that support the findings of this study are available from the corresponding author, upon reasonable request.
