# Supplementary material for: Vapor growth of V-doped MoS2 monolayers with enhanced B-exciton emission and broad spectral response
Source: Front Optoelectron. 2023 Dec 7;16(1):42. doi: 10.1007/s12200-023-00097-w (PMC10703759; doi:10.1007/s12200-023-00097-w)
Supplement: Supplementary file 1 — Supplementary file1 (PDF 587 KB) [file 12200_2023_97_MOESM1_ESM.pdf]

Supporting Information for

Vapor Growth of V-Doped MoS_2 Monolayers with Enhanced B-Exciton Emission and Broad Spectral Response

Biyuan Zheng[1], Xingxia Sun[1], Weihao Zheng[2], Chenguang Zhu[1], Chao Ma[1], Anlian Pan[1], Dong Li[1]*, and Shengman Li[1]*

[1]Key Laboratory for Micro-Nano Physics and Technology of Hunan Province, State Key Laboratory of Chemo/Biosensing and Chemometrics, Hunan Institute of Optoelectronic Integration, College of Materials Science and Engineering, Hunan University, Changsha, 410082, P.R. China.

[2]College of Advanced Interdisciplinary Studies & Hunan Provincial Key Laboratory of Novel Nano Optoelectronic Information Materials and Devices, National University of Defense Technology, Changsha, Hunan 410073, P.R. China.

Emails: liidong@hnu.edu.cn; smli@hnu.edu.cn

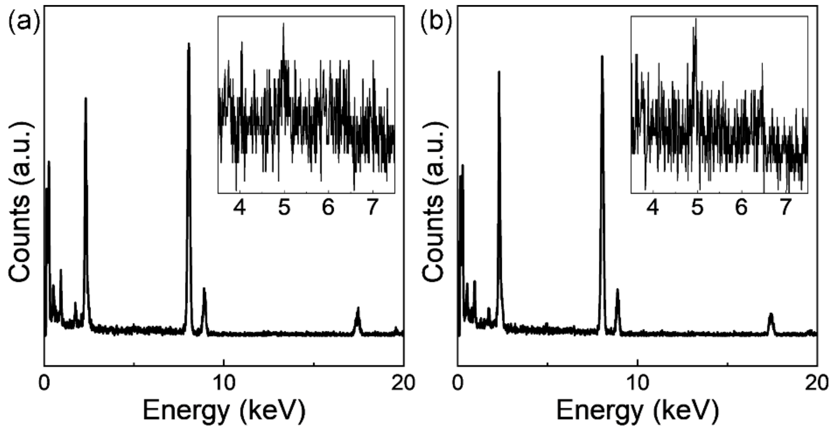

Fig. S1. (a,b) TEM-EDX profiles of the (a) $V_{0.02}Mo_{0.98}S_2$ and (b) $V_{0.05}Mo_{0.95}S_2$ monolayers.

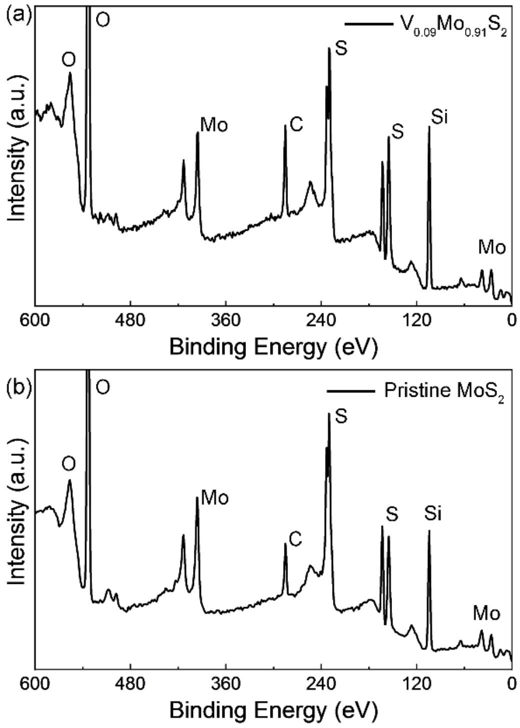

Fig. S2. XPS survey spectra of the (a) $V_{0.09}Mo_{0.91}$ and (b) pristine MoS_2 monolayers.

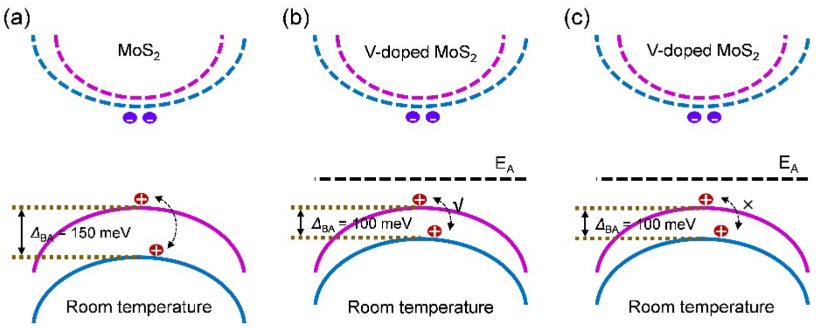

Fig. S3. Illustration of the A- and B-excitons of pristine MoS_2 and V-doped MoS_2. E_A is the V introduced accept impurity state.

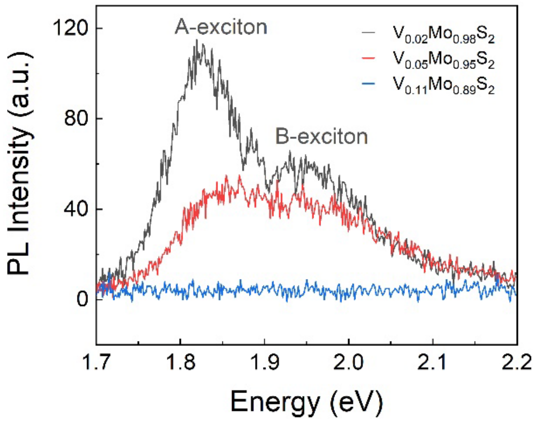

Fig. S4. The PL spectra of the V-doped MoS_2 monolayers with different V composition.

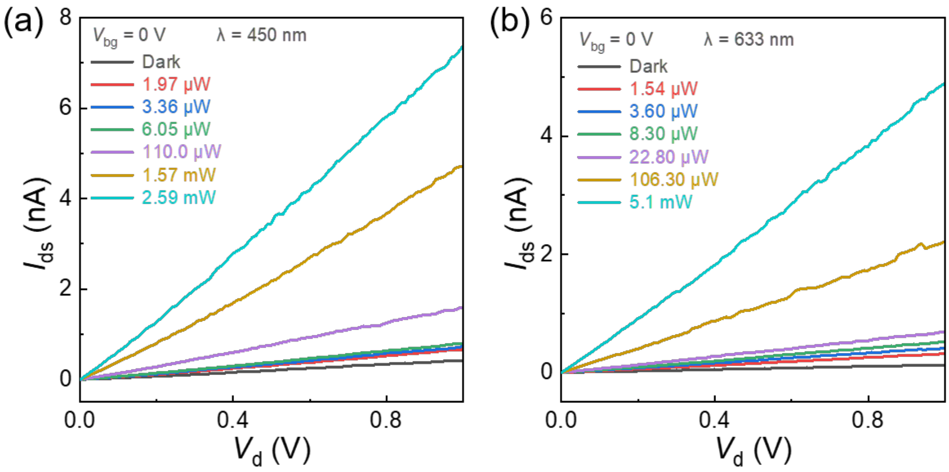

Fig. S5. Photoconductive properties of the V-doped MoS_2 monolayer device under illumination by (a) 450 and (b) 633 nm lasers.

Table S1. Comparison of performances of the photodetectors based on V-doped MoS_2 monolayer and other similar CVD 2D materials.

Materials	Visible Laser	Photoresponsivity	Detectivity	Near-Infrared Laser	Photoresponsivity	Detectivity	Reference
MoS_2	515 nm	1.1 mA/W at 1.5 V	-	-	-	-	44
MoS_2	632 nm	15.6 A/W at 3 V	-	-	-	-	45
MoS_2	532 nm	8.4 mA/W at 0.6 V	1.74×10^{10} Jones	808 nm	-	-	47
WSe_2	550 nm	0.171 A/W at 2 V	10^{12} Jones	-	-	-	47
GQDs/WSe_2/Si	740 nm	0.707 A/W at -3 V	4.51×10^{9} Jones	-	-	-	48
MoS_2/Graphene	-	-	-	1550 nm	1.3 A/W at -3 V	-	49
SnS_2/WSe_2	520 nm	108.7 mA/W at 5 V	4.71×10^{10} Jones	-	-	-	50
In_2S_3/MoS_2	450 nm	4.47 A/W at 1 V	1.07×10^{9} Jones	830 nm	3.2 mA/W at 1.0 V	3.02×10^{6} Jones	51
$Mo_xRe_{1-x}S_2$	405 nm	4.3 mA/W at 1 V	4.8×10^{6} Jones	-	-	-	52
**V-doped MoS_2**	**450 and 633 nm**	**0.23 and 0.24 A/W at 1 V**	**6.59×10^{8} and 1.31×10^{9} Jones**	**980 nm**	**0.14 mA/W at 1 V**	**1.07×10^{6} Jones**	**This work**
